# The Influence of Lymph Node Count on Oncological Outcome of Radical Cystectomy in Chemotherapy Pre-Treated and Chemotherapy-Naïve Patients with Muscle Invasive Bladder Cancer

**DOI:** 10.3390/jcm10214923

**Published:** 2021-10-25

**Authors:** Artur Lemiński, Krystian Kaczmarek, Wojciech Michalski, Bartosz Małkiewicz, Katarzyna Kotfis, Marcin Słojewski

**Affiliations:** 1 Department of Urology and Urological Oncology, Pomeranian Medical University, Powstańców Wielkopolskich 72, 70-111 Szczecin, Poland; k.kaczmarek.md@gmail.com (K.K.); mslojewski@gmail.com (M.S.); 2 Department of Urological Cancer, Maria Sklodowska-Curie National Research Institute of Oncology (MSCNRIO), Roentgena 5, 02-781 Warsaw, Poland; Wojciech.Michalski2@pib-nio.pl; 3 Department of Minimally Invasive and Robotic Urology, University Center of Excellence in Urology, Wroclaw Medical University, Borowska 213, 50-556 Wrocław, Poland; bartosz.malkiewicz@umed.wroc.pl; 4 Department of Anesthesiology, Intensive Therapy and Acute Intoxications, Pomeranian Medical University, Powstańców Wielkopolskich 72, 70-111 Szczecin, Poland

**Keywords:** bladder cancer, neoadjuvant chemotherapy, lymphadenectomy, survival analysis

## Abstract

Radical cystectomy (RC) with pelvic lymph node dissection (PLND) remains the mainstay of treatment for muscle-invasive bladder cancer (MIBC). The extent of PLND and number of removed lymph nodes (LNs) have been associated with improved staging and survival outcomes in several series of RC patients. Neoadjuvant chemotherapy (NAC) has become standard of care for cisplatin-eligible patients qualified to RC, yet few studies on PLND stratified cases according to the receipt of NAC. We aimed to address this issue and reevaluate the prognostic value of PLND nodal yields in series of patients who underwent RC on the verge of the NAC era. This single-center, retrospective, clinical follow-up study enrolled 439 consecutive patients, out of whom 83 received NAC. We analyzed survival outcome of RC according to the number of removed nodes between NAC and non-NAC subgroups. We found PLND thresholds of 10 and 15 LNs prognostically meaningful in our study cohort, and this association was particularly pronounced in the non-NAC subgroup. Higher numbers of LNs provided a 25% reduction in risk of all-cause mortality and correspondingly correlated with up to a 14% increase in 3-year overall survival. The receipt of NAC diminished the benefit of adequate PLND, as the number of retrieved LNs was not associated with survival in the NAC-RC cohort. Given the limitations of our study, additional research is needed to verify these findings.

## 1. Introduction

Radical cystectomy (RC) with pelvic lymph node dissection (PLND) remains the mainstay of treatment for muscle invasive bladder cancer (MIBC). There is substantial evidence concerning the diagnostic and therapeutic role of PLND with anatomical extent of dissection, number of resected lymph nodes (LNs) and lymph node density associated with improved staging, better local disease control, and improved oncological outcome of RC [1,2]. Several authors proposed different anatomical boundaries of PLND along with thresholds of the number of lymph nodes as surrogate markers of oncologically adequate dissection, yet the consensus on the extent of PLND at the time of radical cystectomy has not been established, and the procedure remains underutilized [3,4]. Merely half of patients undergoing RC in the United States over the last two decades underwent an adequate PLND yielding 10 or more lymph nodes [5,6]. Further controversies arise from a recent prospective randomized trial of standard versus extended PLND in an RC cohort (LEA AUO AB 25/02), which has fallen short of corroborating the survival benefit associated with extended lymph node dissection [7]. Moreover, the current standard of care includes administration of neoadjuvant chemotherapy (NAC) in patients eligible for cisplatin, hence up to 60% of patients undergoing contemporary RC receive platinum-based combination NAC [8]. Administration of NAC provides up to 50% of downstaging to <ypT2N0, with complete remissions achievable in one-third of patients [9]. This evolving landscape of treatment for MIBC raises questions whether the number of lymph nodes retrieved during PLND remains an effective prognosticator in patients undergoing RC in the NAC era.

The objective of our study was to reassess the influence of the number of lymph nodes retrieved at the time of surgery in patients with muscle-invasive bladder cancer on long-term prognosis after radical cystectomy in the neoadjuvant chemotherapy era.

## 2. Materials and Methods

**Figure 1:**
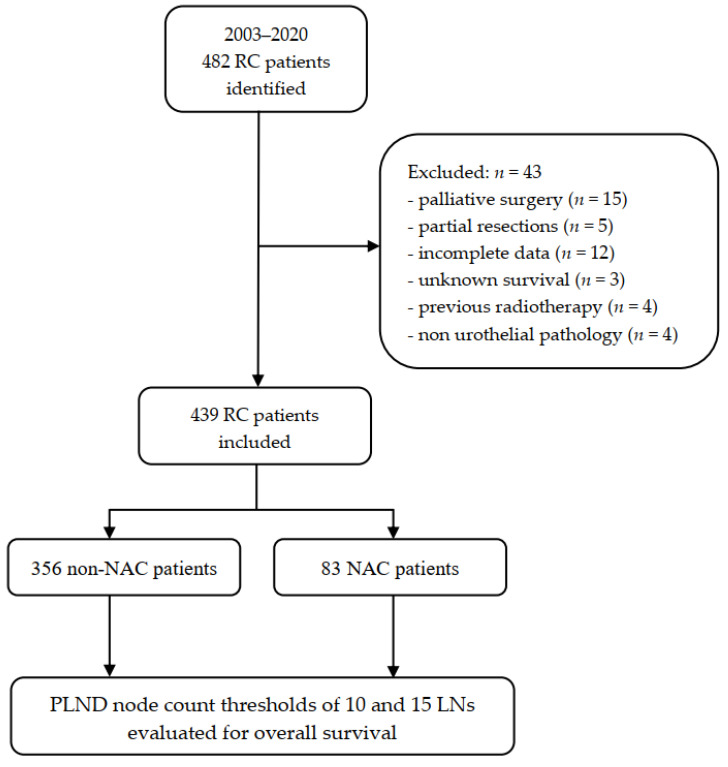
Flowchart of the study. NAC: neoadjuvant chemotherapy; RC: radical cystectomy; PLND: pelvic lymph node dissection.

This single-center non-randomized clinical follow-up study was exempt from further review by the Institutional Review Board (Bioethical Committee) of the Pomeranian Medical University, Szczecin, Poland (protocol number KB-0012/94/08/2021/Z) and was conducted with respect to regulations set forth by the Declaration of Helsinki. All involved patients were routinely consented for participation in research, namely for use of their anonymized treatment data collected during hospital stay for further scientific purposes. We included 482 consecutive patients who underwent RC and PLND due to pathologically confirmed MIBC at the Department of Urology and Urological Oncology of the Pomeranian Medical University, Szczecin, Poland between 2003 and 2020. The neoadjuvant chemotherapy has been routinely offered to patients qualified for RC from 2017. Since then, the uptake of NAC has averaged 55.7% and it was administered to 88 patients until the end of 2020. The remaining part of the study population underwent an upfront cystectomy with eventual adjuvant treatment depending on the final pathologic stage (non-NAC group *n* = 394). The standard PLND template included external and internal iliac, obturator, and common iliac LNs until the crossing of the ureter. This could be further extended cranially to the aortic bifurcation and medially to the presacral area at the discretion of the surgeon. Lymphatic tissue handling was carried out according to standardized protocol for uropathology. Tissue packets undergone fixation in 10% neutral buffered formalin. LNs were identified manually and submitted separately for histological processing. Moreover, additional microscopic examination of remaining adipose tissue was carried out for identification of residual LNs not found on palpation. Only LNs with identified capsule and sinus were counted. We evaluated the influence of the number of LNs retrieved during RC on survival in the entire cohort and compared outcomes between NAC and non-NAC patients. We chose to evaluate two cut-off thresholds representing an “adequate” PLND: the classical approach of 10 LNs and the more extensive approach of 15 LNs, as was previously reported in the literature. Hence, the cohort was divided into subgroups of <10 LNs vs. ≥10 LNs and <15 LNs vs. ≥15 LNs accordingly [2,5,6]. We excluded patients with metastatic disease, those who underwent cystectomy for palliative indications, partial bladder resections, patients with previous pelvic radiotherapy, those with incomplete clinical data, unknown survival status, and non-urothelial pathology from analysis (Figure 1). Data were checked for internal consistency. Descriptive statistics including mean ± standard deviation (SD) and median (interquartile range—IQR) were provided for normally distributed and skewed data, respectively. Single variables were compared using an independent T-test for parametric variables, and a Mann–Whitney U-test and Kruskal–Wallis H-test for non-parametric variables. The survival probabilities over time were presented with Kaplan–Meier survival estimates and univariate Cox models. The survival curves of different groups were compared using the log-rank test. Multivariable Cox proportional hazards models were applied to examine the impact of prognostic factors on survival. These included age at the time of surgery, gender, severity of comorbidities reflected by the American Society of Anesthesiologists (ASA) score, type of surgical approach to RC (open—ORC vs. laparoscopic—LRC), pathological stage, and surgical margin status. Assessment of the proportional hazards assumption of the final multivariable models was carried out using scaled Schoenfeld residuals with time, to test for independence between residuals and time. The results of Cox proportional hazard models were presented as hazard ratios (HR) along with their 95% confidence intervals (CIs). We refrained from assessment of post-hoc power in case of statistically nonsignificant results due to the intrinsic limitations of this method in retrospective studies [10,11,12]. Instead, we chose to perform a confidence interval analysis to inform of the possibility of inadequate sample size [13]. We considered the *p* value < 0.05 statistically significant and all *p* values were two-sided. All tests were performed with Statistica software, version 13.5 (StatSoft, Inc., Tulsa, OK, USA).

## 3. Results

After applying exclusion criteria, the study group comprised 439 patients (356 non-NAC and 83 NAC patients). The median follow-up time was 20.73 months IQR (9.467–52.233) and there were no significant differences between the subgroups: non-NAC: 21.33 months IQR (9.25–64.03) versus NAC: 19.33 months IQR (10.43–33.10). Overall, we identified 111 patients (25.3%) who did not receive an adequate PLND of at least 10 LNs. There was a higher proportion of women with inadequate PLND, 38.14%, as compared to 21.6% of men (*p* = 0.017), whereas the median number of lymph nodes retrieved did not differ significantly between sexes. Applying a higher threshold of 15 LNs resulted in subgroups of 245 (55.8%) and 194 patients (44.2%) with nodal yields of <15 LNs and ≥15 LNs, respectively. There were 116 (26.4%) LRCs in our study population, and we found a significantly higher proportion of these procedures with lymphadenectomy yield of less than 15 LNs, 63.8%, compared to 52.94% for ORCs (*p* = 0.043). As LRC is a procedure with a long learning curve, we found the median LN number of initial procedures significantly lower, as compared to contemporary cases (9 vs. 15.5 for years 2016 and 2020, respectively; *p* = 0.007).

We found no further differences in demographic data, comorbidities, local stage distribution, lymph node metastasis, positive surgical margins, and exposure to NAC among the study subgroups. The study population characteristics are summarized in Table 1.

**Figure 2:**
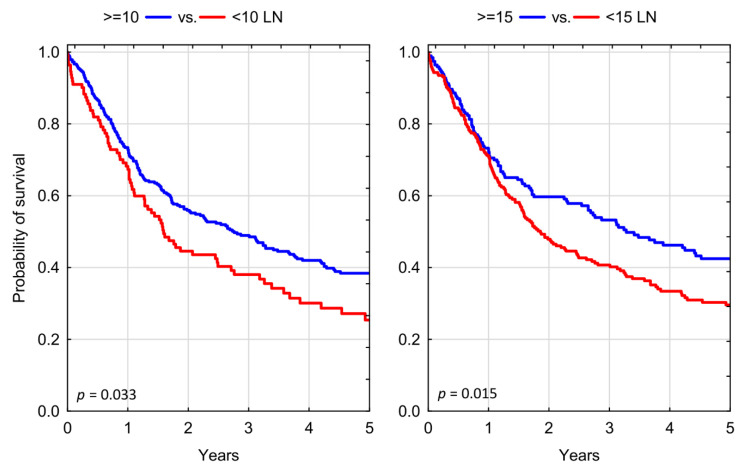
Kaplan–Meier analysis of overall survival in patients who underwent radical cystectomy stratified according to different thresholds of adequate lymph node dissection; LN: lymph nodes.

We found both the evaluated lymph node number cut-off thresholds of 10 and 15 LNs prognostically valid in our study cohort. Patients who received an adequate PLND of at least 10 LNs had a significantly decreased risk of all-cause mortality, as compared to those with inadequate dissection HR = 0.748 (95% CI: 0.577–0.970); *p* = 0.028. Testing a higher LN count threshold, we found a similar reduction in risk of all-cause mortality for those who received a PLND yielding ≥ 15 LNs, as compared to those with less than 15 LNs retrieved HR = 0.742 (95% CI: 0.583–0.945); *p* = 0.015 (Table 2). Expectedly, three-year overall survival (OS) rates were significantly higher in patients who had more LNs resected at the time of RC, with OS of 38.06% for <10 LNs vs. 48.93% for ≥10 LNs; *p* = 0.033; and OS of 40.69% for <15 LNs vs. 53.23% for >15 LNs; *p* = 0.015, respectively (Figure 2).

**Figure 3:**
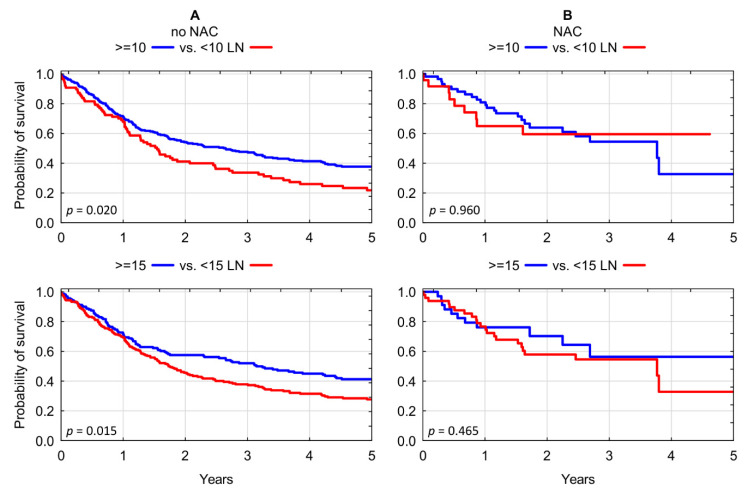
(**A**) Kaplan–Meier analysis of overall survival in chemotherapy-naïve patients who underwent radical cystectomy stratified according to lymphadenectomy yield of <10 LNs vs. ≥10 LNs and <15 LNs vs. ≥15 LNs. (**B**) Kaplan–Meier analysis of overall survival in chemotherapy pre-treated patients who underwent radical cystectomy stratified according to lymphadenectomy yield of <10 LNs vs. ≥10 LNs and <15 LNs vs. ≥15 LNs. LN: lymph nodes; NAC: neoadjuvant chemotherapy.

The above findings were further evaluated in a subgroup analysis. Chemotherapy-naïve patients consistently demonstrated significant overall survival benefit associated with more extensive lymph node dissection for each of the evaluated thresholds: 3-year OS of 33.69% for <10 LNs vs. 47.65% for ≥10 LNs; *p* = 0.020; and OS of 37.84% for <15 LNs vs. 51.99% for >15 LNs; *p* = 0.015) (Figure 3A). In contrast, we found no significant association between lymphadenectomy yield of RC and overall survival in recipients of neoadjuvant chemotherapy: 3-year OS of 59.51% for <10 LNs vs. 54.51% for ≥10 LNs; *p* = 0.959; and OS of 54.63% for <15 LNs vs. 56.36% for >15 LNs; *p* = 0.465) (Figure 3B and Table 3).

## 4. Discussion

Pelvic lymph node dissection remains an integral part of radical cystectomy aiming at accurate pathological staging of cancer and eradication of micrometastatic disease. Significant evidence exists on improvement of cancer control and outcomes of RC through an adequate PLND, hence it is advocated for all patients undergoing RC [14]. Our study, covering 16 years of tertiary academic center experience, revealed that nearly three quarters of patients undergoing RC had an adequate PLND of at least 10 LNs. This proportion is significantly higher than reported by Cole et al., who analyzed data from the Surveillance, Epidemiology and End Results (SEER) database and found that only 45.2% of patients had adequate PLNDs at the time of RC [5]. Although the receipt of adequate PLND was shown to increase over time, the newest data to be analyzed (2008–2010) demonstrated approximately 39% of patients with suboptimal lymphadenectomy. Likewise, von Landenberg et al. retrieved data from the National Cancer Database (NCDB) and found only 52.55% of PLNDs yielding ≥ 10 LNs in patients subjected to RC between 2004 and 2012 [6]. Outcomes comparable to our series were reported by Monaghan et al., who found 74.4% of adequate PLNDs in their NCDB analysis; however, the authors assumed an uncommon and liberal definition of adequate lymphadenectomy, with a cut-off value of just 8 LNs [15].

There is a well-documented problem of inferior outcome of MIBC treatment in women, which despite many efforts remains unsolved, and its underlying mechanisms only partly understood [16]. The results of our study highlight a substantial gender disparity in receipt of adequate lymphadenectomy, with women being nearly twice less likely to receive PLND of more than 10 lymph nodes. A similar observation was made by Cole et al., who revealed that women were indeed less likely to undergo an adequate PLND compared to men: OR 0.87 (0.79–0.97, *p* = 0.01); however, this finding was not confirmed in multivariable analysis [5]. Additional evidence was provided by Siegrist et al. in their retrospective observational study conducted among 1142 patients who underwent RC at Memorial Sloan-Kettering Cancer Center, New York, USA. The authors found that women were significantly less likely to undergo a PLND at the time of RC than men without a clear underlying cause [17]. One plausible explanation for this phenomenon is that a certain proportion of women could have undergone previous pelvic surgery, hampering the possibility of successful LN harvesting. Nonetheless, we were unable to corroborate this notion in our cohort. Notwithstanding, in our study, the median number of LNs retrieved in women was not significantly lower than in men, and female patients had equivalent survival outcome of RC.

Patients who underwent laparoscopic RC had significantly lower median lymph node yield than those treated with the conventional open technique (12 vs. 14, *p* = 0.0094). Consequently, there was a significantly higher proportion of LRCs in the subgroup of patients with a nodal yield of less than 15. The LRC has been performed in our department since 2016, and as a complex pelvic surgery it required a significant caseload to master the technique. The median lymph node number of initial procedures was significantly lower compared to contemporary cases (9 vs. 15.5 for years 2016 and 2020, respectively; *p* = 0.0065), hence the lower median nodal yield of LRC is likely to represent a learning curve phenomenon [18]. The median lymph node count of our laparoscopic cohort is comparable to other early LRC series [19,20,21]. Interestingly, our ORC group had higher nodal yield than most published Polish studies comparing laparoscopic and conventional approaches to RC [20].

We found both lymphadenectomy thresholds of 10 and 15 lymph nodes prognostically meaningful in our study cohort, and this association was particularly pronounced in the non-NAC subgroup. The higher numbers of LNs retrieved provided a 25% reduction in risk of all-cause mortality. Moreover, higher node counts were correlated with an increase in the 3-year overall survival rate in the range of 10% to 14% for both tested thresholds of 10 and 15 LNs. This finding is consistent with numerous observational and database studies published so far, including the pioneering paper from Herr et al. [2,5,22].

Neoadjuvant chemotherapy has become standard of care for patients qualified to RC. There are several cytotoxic combinations used for this indication, largely developed around cisplatin, which shows good activity against urothelial cancer. In principle, NAC apart from local disease downstaging, may serve a similar purpose to lymph node dissection, eliminating potential micrometastatic spread of cancer [23]. This phenomenon, which may diminish the therapeutic role of lymphadenectomy, has been extensively studied in other solid tumors, including ovarian cancer and breast cancer, where administration of NAC has led to a significant decline in the number of axillary lymph node dissections [24,25,26]. The results of our study indicate that patients who received NAC may not benefit oncologically from more extensive LN dissection, as the number of retrieved LNs is no longer associated with survival in this cohort. There is limited evidence documenting this relationship in the available literature, as most studies on PLND have not stratified cases according to the receipt of NAC. Findings similar to our study were reported by von Landenberg et al., who analyzed the records of 16,505 cystectomy cases from the NCDB database. The authors tested similar node count cut-offs of 10 and 15 LNs, and found that the benefit associated with higher nodal yield was limited to patients treated with upfront RC, whereas patients pre-treated with NAC experienced no survival advantage with more extensive PLND [6]. Given that extended pelvic lymphadenectomy is not free from complications and may contribute to morbidity of RC, it seems an attractive possibility to constrain the lymph node dissection in recipients of NAC [27]. This could help limit the inherent collateral damage of PLND, likely without sacrificing the oncological outcome. Additionally, taking into account controversies regarding the shortcomings of PLND in laparoscopic RC, as well as reports of early distant metastatic recurrences after LRCs, it seems reasonable to prefer patients pre-treated with NAC as candidates for minimally invasive RC [28,29].

There are several limitations of our study that need to be acknowledged. This is a single center, non-randomized, retrospective comparison of survival outcomes of different lymphadenectomy yields between a contemporary NAC-RC cohort and a predominantly historical cohort of upfront RCs. There are several factors that may confound the analysis of survival, including the probability of patient selection, evolution of medical care, perioperative support, or timing and delivery of adjuvant treatment. Additionally, we analyzed the number of lymph nodes retrieved as a surrogate marker of the extent of PLND, rather than true anatomical templates of dissection. The number of lymph nodes is an estimate of the quality of PLND, and albeit widely employed in different studies, it is prone to error, which may originate from the surgical technique, tissue handling and fragmentation, pathologic evaluation, and influence of previous treatment [3]. Moreover, as the routine administration of NAC was introduced in our setting only four years ago, the corresponding NAC-RC cohort comprised only 83 patients, compared to 356 upfront RCs. It also remains heterogenous in terms of cytotoxic regimens with predominant dose dense MVAC and gemcitabine-cisplatin, along with numbers of treatment cycles delivered. Lastly, as data regarding the cause of death were not always available, we chose to evaluate overall survival only, which may introduce additional bias due to concurrent mortality, potentially significant in this population.

## 5. Conclusions

This single-center retrospective study indicates a lack of association between adequate PLND and overall survival in patients undergoing multidisciplinary treatment of MIBC involving NAC and RC. Nonetheless, patients treated with upfront cystectomy continue to benefit from an adequate PLND. It seems plausible that administration of NAC diminishes the therapeutic benefit of adequate PLND. Given the preliminary character and limitations of our study, further research is needed to verify these outcomes.

## Figures and Tables

**Table 1:** Baseline patients’ characteristics.

		LN Count			LN Count		
Variable		<10	≥10	*p* Value	<15	≥15	*p* Value
Totals, No.		111	328		245	194	
Age, years				0.848			0.953
Mean		64.765	64.593		64.657	64.611	
SD		9.587	7.716		8.501	7.868	
Sex, No.				0.017			0.756
Female		37	60		56	41	
Male		74	268		189	153	
ASA score, No.				0.179			0.768
1		4	13		7	10	
2		101	279		215	165	
3		6	35		23	18	
4		0	1		0	1	
Pathological T stage, No.				0.539			0.948
pT0		22	32		36	18	
pTis/Ta/T1		9	42		25	26	
pT2		18	63		42	39	
pT3		33	113		80	66	
pT4		29	78		62	45	
Pathological N stage, No.				0.759			0.606
pN0		75	228		166	137	
pN+		36	100		79	57	
Surgical margin				0.576			0.906
Negative		96	290		216	170	
Positive		15	38		29	24	
Neoadjuvant chemotherapy, No.				0.567			0.656
No		87	269		196	160	
Yes		24	59		49	34	
Surgical approach				0.375			0.043
Open (ORC)		77	246		171	152	
Laparoscopic (LRC)		34	82		74	42	

ASA score: American Society of Anesthesiologists score; LN: lymph nodes; SD standard deviation; ORC: open radical cystectomy; LRC: laparoscopic radical cystectomy.

**Table 2:** Effect of adequate lymphadenectomy in univariate and multivariate Cox regression analyses for prediction of overall survival, stratified according to the receipt of neoadjuvant chemotherapy.

	HR	95% CI Lower	95% CI Upper	*p*
Univariate analysis: ≥10 vs. <10 LN				
all patients	0.748	0.577	0.970	0.028
no NAC	0.713	0.541	0.941	0.017
NAC	0.980	0.455	2.111	0.959
Multivariate analysis: ≥10 vs. <10 LN				
all patients	0.714	0.549	0.930	0.012
no NAC	0.718	0.542	0.952	0.021
NAC	0.882	0.329	2.364	0.804
Univariate analysis: ≥15 vs. <15 LN				
all patients	0.742	0.583	0.945	0.015
no NAC	0.729	0.564	0.943	0.016
NAC	0.764	0.370	1.577	0.466
Multivariate analysis: ≥15 vs. <15 LN				
all patients	0.754	0.590	0.963	0.024
no NAC	0.759	0.585	0.983	0.037
NAC	0.695	0.296	1.628	0.402

HR: Hazard ratio; NAC: neoadjuvant chemotherapy; LN: lymph nodes; CI: confidence interval.

**Table 3:** Three-year overall survival of patients who underwent radical cystectomy according to the number of dissected lymph nodes.

LN Count	<10	≥10	*p*	<15	≥15	*p*
All patients	38.06%	48.93%	0.033	40.69%	53.23%	0.015
Chemotherapy						
no NAC	33.69%	47.65%	0.020	37.84%	51.99%	0.015
NAC	59.51%	54.51%	0.959	54.63%	56.36%	0.465

NAC: neoadjuvant chemotherapy; LN: lymph nodes.

## Data Availability

Source data available at: https://osf.io/by2c3, accessed date: 24 October 2021.
